# Intrahepatic Cholestasis of Pregnancy: Role of Baby’s Sex on Itch Severity and Bile Acid Levels

**DOI:** 10.7759/cureus.14089

**Published:** 2021-03-24

**Authors:** Samantha Bartolone, Harvey N Mayrovitz

**Affiliations:** 1 Obstetrics and Gynecology, Dr. Kiran C. Patel College of Osteopathic Medicine, Nova Southeastern University, Davie, USA; 2 Medical Education, Dr. Kiran C. Patel College of Osteopathic Medicine, Nova Southeastern University, Davie, USA

**Keywords:** intrahepatic cholestasis of pregnancy, bile acid, itching in pregnancy, fetal sex differences, gestational disorder, liver disorder, gestational disorder of liver

## Abstract

Background

Intrahepatic cholestasis of pregnancy (ICP) is the most common liver disorder of pregnancy. It occurs when bile flow from the liver is obstructed, causing bile acid to accumulate. ICP usually presents with itching that lasts for the remainder of pregnancy. Untreated ICP has been shown to increase the incidence of adverse outcomes for the baby, including respiratory distress, meconium aspiration, neonatal intensive care unit hospitalization, and stillbirth. The probability of these events increases when bile acid levels are greater than 40 mmol/L. The current standard of care for ICP patients is a combination of ursodeoxycholic acid by mouth and early delivery, as the risk of stillbirth due to ICP increases in the last weeks of pregnancy. ICP has been linked to preeclampsia and gestational diabetes; studies have shown that the occurrence of both diseases is greater when the baby is male. The purpose of this study was to investigate if the occurrence, timing of diagnosis, peak bile acid levels, or severity of itching is dependent on the baby’s sex in mothers who had ICP.

Methods

An online question-set (survey) was offered to women who had had ICP and were members of three different online support groups.

Results

A total of 1,502 women responded for a total of 2,289 documented ICP pregnancies, in which there were 1,059 female babies and 1,230 male babies. Based on chi-square analysis of differences in frequencies, no difference was found between pregnancies with male versus female babies regarding the occurrence, timing of diagnosis, peak bile acid levels, or severity of itching in the evaluated population. However, surprisingly, the findings revealed that first-trimester diagnosis of ICP was made in 5.6% of the pregnancies, and there were 30 (1.8%) cases of ICP diagnosed in the absence of itching.

Conclusions

The findings suggest that the baby’s sex does not impact the occurrence, timing of diagnosis, peak bile acid levels, or itch severity in ICP. The findings that 5.6% of the ICP pregnancies in the present study were diagnosed in the first trimester and 1.8% were diagnosed without the presence of itching reinforce the need to investigate these less common presentations. More studies are needed to determine if these findings are consistent across other populations.

## Introduction

Intrahepatic cholestasis results from obstruction of bile flow. Some causes of intrahepatic cholestasis include progressive familial intrahepatic cholestasis, primary biliary cholangitis, hepatitis, metastatic disease, certain medications, and intrahepatic cholestasis of pregnancy (ICP) [1]. ICP is the most common gestational disorder of the liver, with an occurrence that varies by ethnicity and geographic region (0.1-15.6%) [2]. Others include acute fatty liver disease of pregnancy, hemolysis with elevated liver enzymes and low platelets, and liver dysfunction from preeclampsia [3]. ICP usually manifests itself during the third trimester, or late in the second trimester [4], and most commonly presents with itching that lasts for the remainder of pregnancy. Less common symptoms include jaundice, right upper quadrant pain, pale stool, and dark urine. The implications for the fetus, however, are more serious. Untreated ICP has been shown to increase the incidence of adverse outcomes for the baby, including respiratory distress, meconium aspiration, and neonatal intensive care unit hospitalization due to both pre-term delivery and the disease itself [5]. The current standard of care for ICP patients is a combination of ursodeoxycholic acid by mouth and early delivery between weeks 34 and 38, depending on peak bile acid levels and individual patient circumstance, as the risk of stillbirth due to ICP increases in the last weeks of pregnancy [6,7]. Since the disease is relatively rare [8] and symptoms often nonspecific, it is important for healthcare practitioners to be aware of the signs and symptoms of the disease to help reduce or prevent adverse outcomes.

The exact mechanisms causing intrahepatic cholestasis to occur during pregnancy are still unknown; however, two adenosine triphosphate binding cassette genes (*ABCB4* and *ABCB11*) have been identified as contributors in some women [9]. The genetic profile of cholestasis continues to be researched extensively. Certain genetic mutations, in addition to other still unknown factors, cause a rise in serum bile acids, which is currently the major diagnostic component of ICP [6].

Current guidelines state that a diagnosis of ICP can be made if serum bile acid levels are above 10 mmol/L. When levels reach 40 mmol/L, the case is considered severe and risk of adverse outcomes is increased [10,11]. When levels reach 100 mmol/L, the risk of adverse fetal outcomes is increased further, with a 3.44% risk of stillbirth [12]. Although the diagnostic criteria are focused on bile acid levels, other liver function parameters such as aspartate aminotransferase (AST), alanine aminotransferase (ALT), and bilirubin are used to obtain a fuller picture and to rule out cases in which other liver diseases are present [13].

Sex differences in pregnancy outcomes and the development of other gestational disorders have been observed [14], providing possible clues to their pathogenesis. However, it is unknown if ICP also presents differently depending on the baby’s sex. The purpose of this study was to investigate whether there is a correlation between the baby’s sex, and the occurrence of ICP, timing of diagnosis, peak bile acid levels, or severity of itching. This research will add to the general body of knowledge on cholestasis of pregnancy and help in the formulation of new research questions.

## Materials and methods

Subjects

To obtain the needed data, a survey was designed and approved by Nova Southeastern University Institutional Review Board. The survey was posted to three existing online (Facebook) support groups. In total, the number of members in the three support groups together was around 20,000. Overlap among the three groups is suspected, with many women assumed to be a part of two or three groups. The members of the groups include women who have had ICP in previous pregnancies, women who are newly diagnosed, women who are concerned about having ICP, and family members or friends of those affected.

Survey questions

A concise survey consisting of 10 questions was posted for the women to answer. The questions were designed to solicit information regarding the number of male and female babies a woman has had, the number of times she has experienced ICP with each sex, and the timing of diagnosis, peak bile acid levels, and itch severity with each of her ICP pregnancies. The questions are given in Table 1.

**Table 1:** Survey questions presented to members of the three Facebook support groups.

Questions	
(1) How many *male* biological children do you have? (Include children when you did *and* did not have ICP)	
(2) With how many of your *male* children were you diagnosed with ICP?	
(3) At what week (approximately) were you diagnosed with each of your *male* ICP babies?	Options: before 13 weeks, 14-27 weeks, 28-40 weeks, do not remember
(4) What was the *highest* bile acid level you remember with each of your *male* ICP babies? (micromol/L)	Options: 10-40, 41-100, >100, do not remember
(5) What was the severity of your itching with each of your *male* ICP babies?	Options: none, mild, moderate, severe
(6) How many *female* biological children do you have? (Include children when you did *and* did not have ICP)	
(7) With how many of your *female* children were you diagnosed with ICP?	
(8) At what week (approximately) were you diagnosed with each of your *female* ICP babies?	Options: before 13 weeks, 14-27 weeks, 28-40 weeks, do not remember
(9) What was the *highest* bile acid level you remember with each of your *female* ICP babies? (micromol/L)	Options: 10-40, 41-100, >100, do not remember
(10) What was the severity of your itching with each of your *female* ICP babies?	Options: none, mild, moderate, severe

All data were collected anonymously via Google forms. The survey yielded a total of 1,502 responses. Data were analyzed using Microsoft Excel (Version 2016) and the Statistical Package for the Social Sciences (SPSS) software Version 16.0 (SPSS Inc., Chicago, IL, USA). Primary analysis addresses differences in cholestasis presence, timing of diagnosis, peak bile acid levels, and severity of itching as they correlate to the sex of the baby.

Analysis

After correcting for the total number of responses by removing “do not remember” and blank responses, a chi-square test of independence was performed to examine the relationship between the sex of the baby and each of the categories of interest, including occurrence, timing of diagnosis, peak bile acid levels, and itch severity.

## Results

The results are summarized in Tables 2, 3. The total number of pregnancies represented was 3,247, of which 2,289 (70.5%) were impacted by ICP diagnosis. There was no significant difference in occurrence of ICP in pregnancies with male versus female babies. Each participant provided a response regarding week of diagnosis. Of the 2,289 ICP pregnancies recorded, 80.5% (1,843) of them included a response regarding peak bile acid levels and 73.0% included a response regarding itch severity. The relationship between the baby’s sex and each of these variables was not significant, with p > 0.05 in each category. It is interesting to note the 5.6% of ICP pregnancies that were diagnosed before 13 weeks and the 1.8% of ICP pregnancies that were diagnosed in the absence of itching.

**Table 2:** ICP occurrence in pregnancies with male versus female babies. Entries are number of responses and percentages in each category. ICP, intrahepatic cholestasis of pregnancy

	Pregnancies with Males	Pregnancies with Females	Combined
Total number of pregnancies	1738	1509	3247
ICP pregnancies	1230 (70.8)	1059 (70.2)	2289 (70.5)

**Table 3:** Survey responses Entries are number of responses and percentages of responses in each category.

	Pregnancies with Males	Pregnancies with Females	Combined
Week of diagnosis			
Total responses	1230 (100)	1059 (100)	2289 (100)
<13	61 (5.0)	67 (6.3)	128 (5.6)
14-27	336 (27.3)	305 (28.8)	641 (28.0)
28-40	833 (67.7)	687 (64.9)	1520 (66.4)
Peak bile acid level (mmol/L)			
Total responses	977 (100)	866 (100)	1843 (100)
10-40	497 (50.9)	411 (47.5)	908 (49.3)
41-100	299 (30.6)	268 (30.9)	567 (30.8)
>100	181 (18.5)	187 (21.6)	368 (19.9)
Itch severity			
Total responses	878 (100)	793 (100)	1671 (100)
No itch	11 (1.3)	19 (2.4)	30 (1.8)
Mild itch	103 (11.7)	97 (12.2)	200 (12.0)
Moderate itch	257 (29.3)	223 (28.1)	480 (28.7)
Severe itch	507 (57.7)	454 (57.3)	961 (57.5)

## Discussion

The present findings suggest that the sex of the baby is not significantly related to the occurrence, timing of diagnosis, peak bile acid levels, or severity of itching in ICP. Only one other study [15] to our knowledge has addressed the question of whether ICP is more common with male or female babies. That study, which included 48 pregnancies of women with ICP at a single hospital, also found no significant sex difference in the occurrence of ICP. No studies investigating the timing of diagnosis, peak bile acid levels, or itch severity in relation to the baby’s sex have been conducted to our knowledge.

Other researchers have searched for relationships between ICP and other gestational disorders and have shown a linkage to both preeclampsia and gestational diabetes mellitus [16-18]. In both these conditions, disease development has shown predilection for pregnancies with male babies. A recent analysis [19] found that preeclampsia is more common in pregnancies with males when looking at all races combined, with an odds ratio of 1.3. Specific populations have shown to differ, however, with the Japanese population showing a greater risk of preeclampsia with female pregnancies, with a male-to-female risk ratio of 0.92 [20]. In addition to preeclampsia, the risk of developing gestational diabetes mellitus has been found to be greater in pregnancies where the baby is male versus female, with an odds ratio of 1.39 [21,22]. More studies are needed to determine if ICP, like preeclampsia, shows fetal sex differences in specific populations.

It is widely reported that ICP occurs most commonly in the third trimester or late second trimester of pregnancy. The present findings are consistent with this, with 66.4% occurring in the third trimester and 28% occurring in the second trimester. Although there was no sex difference in the timing of these diagnoses, it is noteworthy that 5.6% of ICP pregnancies in the present study were diagnosed in the first trimester. Although cases of severe first-trimester ICP are documented in the literature, the statistical percentage of occurrence has not been investigated to our knowledge. However, one study [23] in Latin America, which investigated the relationship between timing of ICP onset and risk of meconium-stained amniotic fluid in 382 ICP pregnancies, included 1.6% of participants who were diagnosed before 24 weeks. On observation, there have been several anecdotal reports of first-trimester ICP in which a diagnosis of ICP is not suspected by the physician due to the timing of presentation. There have been cases published in the literature of early onset ICP [24]. Some of these cases are linked to fertility treatments and other known risk factors such as multiple gestations, heterozygous *ABCB4* genes, or ovarian hyperstimulation syndrome; however, cases have been reported in the first trimester in the absence of any known risk factors [25,26]. This is important because women who present with early persistent itching may need to undergo testing, and retesting even when bile acid levels are normal, as they can become elevated later. This is evidenced by a study that followed up 10 women who had itching on their hands and feet but normal markers of liver function. Each developed ICP diagnosable by liver function tests between 2 and 20 weeks later [27]. Additionally, there is evidence that early onset ICP is associated with greater risk of adverse outcomes [28]. The survey did not collect information about risk factors; therefore, it is unknown what may have contributed to the development of first-trimester ICP in the survey population.

Although the present findings did not discover a linkage between peak bile acid levels and baby’s sex, there were 30 cases where ICP was diagnosed in the absence of any reported itching. Since the survey did not ask, it is not known what symptoms prompted these women to be evaluated in the absence of itching. While most cases of ICP are diagnosable with symptomatic itching and elevated bile acid levels, a correlation between bile acid levels and severity of itching has not been demonstrated [29]. Furthermore, there is the possibility of subclinical cholestasis in which serum bile acids are elevated but itching is not present, as well as situations where itching is present but bile acid levels are not elevated [27]. Although lysophosphatidic acid levels have been found to correlate with itch severity more than bile acid levels [30], more investigation is needed to better determine how both bile acid levels and itch severity relate to other parameters of the disease.

Our study surveyed women who belonged to three different private Facebook groups - ICP Care/Itchy Moms, ICP Support, and Obstetric Cholestasis. It is possible that the survey was biased by selecting only for women who seek support and education from the Facebook groups. In addition, the survey platform had no safeguard in place to prevent the same woman from multiple submissions. Our survey did not consider maternal age, birth order, multiple pregnancies, or complications including stillbirth. Since the responses were not verified with medical records, the assumption was made that the women were able to accurately recall their week of diagnosis and bile acid levels, introducing possible error. A few respondents commented that their bile acid levels were not measured due to lack of protocols at the time of their pregnancies. Some answered with “do not remember” or left the question blank. Thus, some respondents may have answered the survey incompletely according to their individual situation. These responses were not included in the total number of responses in each category. This should not have greatly affected the results of the study, and most participants were able to provide responses to the questions presented.

## Conclusions

The findings suggest that the baby’s sex does not impact the occurrence, timing of diagnosis, peak bile acid levels, or itch severity in ICP. Other studies are needed to determine if this is consistent across other populations. Since it was found that 5.6% of the ICP pregnancies in the present study were diagnosed in the first trimester, more investigation is needed to understand what risk factors these mothers had. Therefore, more studies of cases of first-trimester ICP would be useful. Our finding of 1.8% of the ICP pregnancies without the presence of itching suggests that there could be women who are not aware they have this disease and therefore are not diagnosed or treated. This helps to reinforce the idea that screening in the absence of symptoms could be useful. Overall, these study results add to the general body of knowledge pertaining to ICP and will help in the formulation of new research questions.
